# Identification of Subsets of Enteroaggregative *Escherichia coli* Associated with Diarrheal Disease among Under 5 Years of Age Children from Rural Gambia

**DOI:** 10.4269/ajtmh.16-0705

**Published:** 2017-08-07

**Authors:** Usman N. Ikumapayi, Nadia Boisen, Mohammad J. Hossain, Modupeh Betts, Modou Lamin, Debasish Saha, Brenda Kwambana-Adams, Michel Dione, Richard A. Adegbola, Anna Roca, James P. Nataro, Martin Antonio

**Affiliations:** 1 Vaccines and Immunity Theme, Medical Research Council Unit The Gambia, Banjul, The Gambia;; 2 Disease Control and Elimination Theme, Medical Research Council Unit The Gambia, Banjul, The Gambia;; 3 Department of Microbiological Surveillance and Research, Statens Serum Institut, Copenhagen, Denmark;; 4 Faculty of Epidemiology and Population Health, London School of Hygiene and Tropical Medicine, London, United Kingdom;; 5 Centre for Vaccine Development, University of Maryland School of Medicine, Baltimore, Maryland;; 6 Department of Pediatrics, University of Virginia School of Medicine, Charlottesville, Virginia;; 7 Faculty of Infectious and Tropical Diseases, London School of Hygiene and Tropical Medicine, London, United Kingdom;; 8 Microbiology and Infection Unit, Warwick Medical School, University of Warwick, Coventry, United Kingdom

## Abstract

Enteroaggregative *Escherichia coli* (EAEC) cause acute and persistent diarrhea, mostly in children worldwide. Outbreaks of diarrhea caused by EAEC have been described, including a large outbreak caused by a Shiga toxin expressing strain. This study investigated the association of EAEC virulence factors with diarrhea in children less than 5 years. We characterized 428 EAEC strains isolated from stool samples obtained from moderate-to-severe diarrhea cases (157) and healthy controls (217) children aged 0–59 months recruited over 3 years as part of the Global Enteric Multicenter Study (GEMS) in The Gambia. Four sets of multiplex polymerase chain reaction were applied to detect 21 EAEC-virulence genes from confirmed EAEC strains that target pCVD432 (aatA) and AAIC (aaiC). In addition, Kirby-Bauer disc diffusion antimicrobial susceptibility testing was performed on 88 EAEC strains following Clinical Laboratory Standard Institute guidelines. We observed that the plasmid-encoded enterotoxin [odds ratio (OR): 6.9, 95% confidence interval (CI): 2.06–29.20, *P* < 0.001], aggregative adherence fimbriae/I fimbriae (aggA) [OR: 2.2, 95% CI: 1.16–4.29, *P* = 0.008], and hexosyltransferase (capU) [OR: 1.9, 95% CI 1.02–3.51, *P* = 0.028] were associated with moderate-to-severe diarrhea among children < 12 months old but not in the older age strata (> 12 months). Our data suggest that some EAEC-virulent factors have age-specific associations with moderate-to-severe diarrhea in infants. Furthermore, our study showed that 85% and 72% of EAEC strains tested were resistant to sulphamethoxazole-trimethoprim and ampicillin, respectively. Sulphamethoxazole-trimethoprim and ampicillin are among the first-line antibiotics used for the treatment of diarrhea in The Gambia.

## INTRODUCTION

Enteroaggregative *Escherichia coli* (EAEC) is an important causative agent of both acute and persistent diarrhea among adults and children worldwide^1^ and it has been among the most common *E. coli* pathotypes causing diarrhea among children less than 5 years of age in some developing countries.^2^ Several outbreaks of EAEC diarrhea have been reported in both developed and developing nations and infants are the most affected.^3–6^ EAEC has been implicated in travelers’ diarrhea^7,8^ and persistent diarrhea among human immunodeficiency virus–infected individuals.^9^ This pathotype was implicated in a massive outbreak of hemolytic uremic syndrome in Germany in 2011.^10^ The clinical presentation of EAEC infection is characterized by watery and mucoid diarrhea with low-grade fever and insignificant vomiting.^11,12^

The pathogenesis of EAEC diarrhea is thought to comprise colonization of the intestinal mucosa, followed by elaboration of enterotoxins and cytotoxins and the release of proinflamatory cytokines from infected epithelial cells,^13,14^ induced by the EAEC adherence factors called aggregative adherence fimbriae (AAF). In addition, EAEC strains characteristically enhance mucus secretion from the mucosa, potentially trapping the bacterium in a bacterium-mucus biofilm.^1^ A distinctive feature of EAEC is its ability to elicit characteristic stacked brick-liked aggregative adherence to HEp-2 or HeLa cells, a test that remains the gold standard to identify this pathotype.^15^ EAEC strains express several genes that may confer virulence and are highly heterogeneous regarding the combination of these virulence genes, which are encoded on the bacterial chromosome or on an EAEC-specific plasmid-designated pAA. The majority of EAEC strains harbor a transcriptional activator of the AraC/XyIS called AggR, which control genes on both the plasmid and the chromosome. Among the genes under AggR control includes those that encode the AAF where at least five variants exist. These genes encoding the major structural pilin subunits are designated as *aggA* (AAF/I), *aafA* (AAF/II), *agg3A* (AAF/III), *agg4A* (AAF/IV), and *agg5A* (AAF/V).^16–18^ Other plasmid-borne potential virulence factors include the EAEC heat-stable enterotoxin 1 (EAST1) (encoded by the *astA* gene),^19^ an anti-aggregation protein called dispersin (encoded by the *aap* gene), and a transporter apparatus for dispersin called Aat (encoded by the *aat* genes). EAEC frequently harbor members of the serine protease autotransporters of Enterobacteriaceae (SPATEs), which have been described as enterotoxins and cytotoxins. The heat-labile enterotoxin/cytotoxin called Plasmid-encoded toxin (Pet)^20^ has been implicated in causing cytotoxic effects on the human intestinal mucosa. Other SPATEs carried by EAEC strains include the cryptic protease called SepA, and the mucinase called Pic (protein involved in intestinal colonization),^21,22^ which is encoded on the chromosome. Other important chromosomal gene that encodes virulence markers include 1) Irp2 (iron repressible high-molecular-weight protein 2) a protein responsible for yersiniabactin biosynthesis and 2) flagellin, which interacts with the epithelial cells, leading to the secretion of an intestinal interleukin-8.^23^ The EAEC genome has been found to be markedly mosaic; thus, the various putative virulence factors are found inconsistently among individual strains, suggesting that some strains considered EAEC may be truly virulent, and others not.^20^

Several studies have shown that EAEC is the most frequently detected *E. coli* pathotype in humans, particularly among children from both developed and developing countries.^24–26^ The Global Enteric Multicenter Study (GEMS) comprised identical case–control studies of moderate-to-severe diarrhea (MSD) among children under 5 years of age at four sites in sub-Saharan Africa and three in south Asia showing high frequency of EAEC.^27^ Although EAEC was not associated with MSD disease in the GEMs study, a subsequent analysis of the association of individual EAEC genes alone and in combination among EAEC isolates from MSD cases and controls of GEMS in Mali by Boisen found that SepA protease was associated with MSD.^28^ In the study presented, we replicated the analysis by Boisen, scoring the presence of 21 putative EAEC virulence factors from 428 EAEC isolates randomly selected among 741 EAEC isolates obtained from diarrheal and nondiarrheal children enrolled in the GEMS study to characterize the virulence genes in children from The Gambia. We analyzed these EAEC virulence genes by age strata (0–11, 12–23, and 24–59 months). Furthermore, antimicrobial resistance patterns were investigated on a 20% of the EAEC strains selected at random.

## MATERIALS AND METHODS

### Patients and enteric pathogens tested.

The study participants were enrolled as part of the 3-year (December 2007 to December 2010) prospective case–control GEMS in The Gambia. The clinical and microbiologic methods for the GEMS have been published.^29^ In GEMS, children aged 0- to 59-month old from a defined census population attending to sentinel health center with sign of MSD were enrolled. One to three healthy controls matched by age, sex, and community were recruited within 14 days after the enrolment of cases.^30^ As part of the main study, stool samples were processed for enteric bacterial pathogens (including *E. coli*, *Salmonella* spp., Shigella spp., Vibrio spp., *Campylobacter* spp., and Aeromonas spp.), Viruses (Sapovirus, Norovirus, and Rotavirus), and parasites (*Giardia lamblia*, Cryptosporidium species, and *Entamoeba histolytica*).^31^ A total of 2,598 children were enrolled in The Gambia and 741 (28.5%) children had EAEC isolated (278 cases and 463 controls).

For this ancillary study, the matching design was not maintained. We randomly selected 428 (58%) of the participants with samples positive for EAEC (157 cases and 271 controls) (Table 1) to conduct genotypic characterization of the 21 virulence factors. Of the 157 cases, 94 (60%) and 63 (40%) account for male and female, respectively, whereas among the controls 151 (56%) account for male and 120 (44%) account for female.

**Table 1:** Baseline information of study population

Demographic factors	Case (*N* = 157) no. (%)	Control (*N* = 271) no. (%)	Total (*N* = 428) no. (%)	OR (95% CI)	*P* value
Age (month)					
0–11	85 (54.1)	132 (48.7)	217 (50.7)	1.2 (0.82–1.88)	0.278
12–23	61 (38.9)	105 (38.8)	166 (38.8)	1.0 (0.65–1.53)	0.982
24–59	11 (7.0)	34 (12.6)	45 (10.5)	0.5 (0.23–1.10)	0.071

CI = confidence interval; OR = odds ratio.

### Testing of 21 enteroaggregative *E. coli* virulence genes.

From an overnight growth on MacConkey agar (Oxoid, Hampshire, United Kingdom), three suspected colonies of *E. coli* (typically lactose fermenting) for each patient were purified and identified as *E. coli* using the biochemical reagent kit Analytical Profile Index 20 E (BioMeriux Ltd, Hampshire, United Kingdom). The resultant confirmed *E. coli* were screened to detect EAEC, ETEC, and EPEC pathotypes using a multiplex polymerize chain reaction (PCR) GEMS protocol.^31^ In this study, we performed monoplex PCR on each isolates that has initially showed presence of EAEC in GEMS *E. coli* multiplex PCR protocol. The target sought for EAEC are the EAEC plasmid-encoded gene *aat*A (primer CVD432F–sequence 5′-CTGGCGAAAGACTGTATCAT-3′and primer CVD432R–sequence 5′-CAATGTATAGAAATCCGCTGTT-3′) and the EAEC chromosomally encoded *aai*C (primer AAIC F–sequence 5′-ATTGTCCTCAGGCATTTCAC-3′ and primer AAIC R-sequence 5′-ACGACACCCCTGATAAACAA-3′), these two loci are known as virulence determinants. EAEC colonies were investigated for the presence of the 21 putative virulence genes using four multiplex PCRs as previously described.^28^ The 21 genes were grouped into four. On each group, multiplex PCR was performed. On group 1 (*sat*, *sepA*, *pic*, *sigA*, plasmid-encoded enterotoxin [*pet*], and *astA*), multiplex PCR master mix was achieved using Qiagen kit (Catalogue no. 206143 [Manchester, United Kingdom]) following the manufacturer’s instructions. Multiplex PCR assay was performed in a final reaction volume of 25 μL consists of 12.5 μL mastemix (MM), 2.5 μL Q-solution, 6 μL primer (MM), 2.5 μL H_2_O, and 1.5 μL DNA template. PCR reaction cycles were as follows: 15 minutes preheating at 95°C at the start, 50 seconds denaturation at 94°C, annealing for 1.5 minutes, and extension at 72°C for 1.5 minutes with 35 cycles returning to step 2. The final extension was 10 minutes at 72°C.

On group 2 (*aatA*, *aggR*, *aaiC*, *aaP*, and ORF3), group 3 (*aafC*, *agg3/4C*, *agg3A*, *aafA*, *aggA*, and *agg4A*), and group 4 (*air*, *capU*, *ailA*, and ORF61) Fementers kit (Catalogue no. K0171 [Paisley, United Kingdom]) was used for the PCR master mix (2X) following the manufacturer’s instructions. Multiplex PCR assay was achieved in a final reaction volume of 25 μL that compose of 12.5 μL (MM), 1 μL (25 mM magnesium chloride), 5 μL primer (MM), 5 μL H_2_O, and 1.5 μL DNA template. PCR reaction cycles were achieved as follows: 2 minutes preheating at 95°C at the start, 50 seconds denaturation at 94°C, annealing at 57°C (58°C for groups 3 and 4) for 1.5 minutes, and extension at 72°C for 1.5 minutes with 35 cycles returning to step 2. The final extension was 10 minutes at 72°C.

Amplifications were performed using Thermocycler (TECHNE Flexigen, Model FFG02FSD, Serial 11733-1 [Paisley, United Kingdom]). Amplified PCR products were analyzed on a 2% agarose gel containing ethidium bromide (final concentration of 0.5 μg/mL in 1 × TBE buffer and visualized on the 2% [w/v] agarose gel under ultraviolet radiation. The gel images were captured digitally with a gel documentation system.

The *E. coli* strains used as controls for detection of the target genes are C1010-00 (*sat*, *sepA*, *agg3/4C*, and *agg4A*), JM221 (*sat* and *aggA*), 042 (*pic*, *pet*, *astA*, *aatA*, *aggR*, *aaiC*, *aap*, *ORF3*, *aafC*, *aaFA*, *air*, *capU*, and *eilA*), 55989 (*sigA*, *agg3A/4C*, and *agg3A*), 63 (*sigA*, *agg3/4C*, and *agg4A*), and 17-2 (*aggA*). GIBCO distilled water (DNase/RNase free, Catalogue no. 10977-035 [Paisley, United Kingdom]) was used as negative control.

### Antimicrobial assay.

For antimicrobial susceptibility testing, 88 EAEC isolates were randomly selected from the 428 EAEC isolates used for the virulence genes assay. Disk diffusion (Kirby Bauer) method for susceptibility testing that allows categorization of bacterial isolates as susceptible, resistant, or intermediate to eight commercially acquired antimicrobial agents which include ampicillin 10 μg, cotrimoxazole 25 μg, tetracycline 30 μg, ceftazidime 30 μg, ciprofloxacin 5 μg, ceftriaxone 30 μg, cefoxitin 30 μg, and nalidixic acid 30 μg (Oxoid) were used. The Clinical Laboratory Standard Institute version M100-S22, Vol. 32, No. 3, 2012, guidelines were followed. *E. coli* ATCC 25922 was used for quality control antibiotic susceptibility assay.

### Ethical consideration.

The GEMS obtained ethical clearance from The Gambian Government/Medical Research Council (MRC) Ethics committee following the scrutiny of the study proposal by the MRC Scientific Coordinating Committee.

### Statistical analyses.

Bivariate analysis was applied to compare prevalence of virulence factors between cases and controls in different age group using STATA 12 reporting odds ratios (ORs) with 95% confidence intervals (CIs). A two-sided *P* value < 0.05 was considered statistically significant.

In addition, we used Classification and Regression Tree (CART) pro-Version 6.0 (Salford Systems, San Diego, CA) software to input 21 factors of interest as binary (present/absent) independent variables. Although, case–control status was input as the binary dependent outcome variable.

### Significance of combinations of EAEC genes.

We generated a virulence factor score (VFS), representing the collective number of virulence loci present in each strain. To consider the combination factors, we used CART analysis (Supplemental Figures 1 and 2), which builds a model in stepwise fashion to yield the combination of factors most strongly associated with the queried outcome. Each branch of a CART output tree ends in a terminal “node”; each observation falls into exactly one terminal node; and each terminal node is uniquely defined by a set of rules, such as having or not having a certain factor.

We considered all genotypic and phenotypic assays performed and considered the association with case status versus control status (Supplemental Figures 1 and 2).

## RESULTS

Among all EAEC strains in cases and controls (*N* = 428), the age and sex distribution were similar among cases and controls except for a lower prevalence of children above 23 months among cases (Table 1). Overall, orf61 (*aar*) was the most commonly detected gene, (69.6%). This was followed by the cryptic ORF3 (64%), *capU* (62%), *aggR* (60.1%), *astA* (51.4%), *eilA* (48.3%), and *aap* (46.3%); the rest of the genes were present in less than 40% of isolates (Table 2). Analysis of the EAEC virulence genes in all age groups together, showed that only four of the 21 genes assayed (*sepA*, *pet*, *astA*, and *capU*) were more prevalent among cases. Prevalence of AAF/I encoded by *aggA* gene was slightly higher in cases than controls (29.9% versus 22.9%) (OR: 1.4, 95% CI: 0.89–2.29, *P* = 0.106) (Table 2). The frequency of other AAF pilin genes, AAF/II (aafA), and AAF/III (agg3A) were low in both cases and controls but slightly high for AAF/IV (agg4A) in cases compared with controls. However, the AAF usher-encoding gene agg3/4C was similar in cases and controls (36.9% versus 35.4%, respectively). Of the five SPATE genes (*sat*, *pet*, *sigA*, *pic*, and *sepA*), prevalence of *sepA* (OR: 1.6, 95% CI: 0.99–2.49, *P* = 0.041) and *pet* (OR: 1.9, 95% CI: 0.97–3.56, *P* = 0.042) genes were higher among diarrhea cases (Table 2).

**Table 2:** Distribution of EAEC virulence genes from case and control children (age 0–59 months)

Gene class		Virulence gene	Case (*N* = 157) no. (%)	Control (*N* = 271) no. (%)	Total (*N* = 428) no. (%)	OR (95% CI)	χ^2^	*P* value
		***aatA***	51 (32.5)	82 (30.3)	133 (31.1)	1.1 (0.71–1.73)	0.2	0.631
		***aggR***	97 (61.8)	161 (59.4)	258 (60.3)	1.1 (0.72–1.69)	0.2	0.628
		***aaP***	77 (49.0)	121 (44.7)	198 (46.3)	1.2 (0.78–1.80)	0.8	0.379
		**ORF3**	106 (67.5)	168 (62.9)	274 (64.0)	1.3 (0.82–1.98)	1.3	0.251
		***capU***	108 (68.8)	158 (58.3)	266 (62.2)	1.6 (1.02–2.45)	4.7	0.031
		***aar***	110 (70.1)	188 (69.4)	298 (69.6)	1.0 (0.66–1.63)	0.1	0.880
pAA	A	***aafC***	7 (4.5)	16 (6.0)	23 (5.4)	0.7 (0.25–1.97)	0.4	0.522
P	D	***agg3/4C***	58 (36.9)	96 (35.4)	154 (36.8)	1.1 (0.69–1.64)	0.1	0.752
L	H	***agg3A***	10 (6.4)	28 (10.3)	38 (9.8)	0.6 (0.25–1.29)	1.9	0.164
A	E	***aafA***	3 (1.9)	15 (5.5)	18 (4.2)	0.3 (0.06–1.20)	3.2	0.071
S	S	***aggA***	47 (29.9)	62 (22.9)	109 (25.5)	1.4 (0.89–2.29)	2.6	0.106
M	I	***agg4A***	15 (9.6)	16 (6.0)	31 (7.2)	1.7 (0.74–3.75)	1.9	0.160
	N							
I	T	***astA***	91 (58.6)	129 (47.6)	220 (51.4)	1.5 (1.00–2.30)	4.3	0.038
D	O	***sat***	29 (18.5)	56 (20.7)	85 (19.9)	0.9 (0.51–1.47)	0.3	0.583
	X	***sepA***	50 (31.9)	62 (22.9)	112 (26.2)	1.6 (0.99–2.49)	4.1	0.041
	I	***pet***	24 (15.3)	24 (8.9)	48 (11.2)	1.9 (0.97–3.56)	4.1	0.042
CH	N	***pic***	55 (35.0)	88 (32.5)	143 (33.4)	1.1 (0.72–1.73)	0.3	0.588
RO	S	***sigA***	18 (11.5)	31 (11.4)	49 (11.5)	1.0 (0.50–1.93)	0.0	0.993
MO		***aaiC***	44 (28.0)	97 (35.8)	141 (32.9)	0.7 (0.44–1.09)	2.7	0.099
SO		***air***	41 (26.1)	57 (21.0)	98 (22.9)	1.3 (0.81–2.15)	1.5	0.227
ME		***eilA***	79 (50.3)	128 (47.2)	207 (48.4)	1.1 (0.75–1.71)	0.4	0.538

CI = confidence interval; EAEC = enteroaggregaive *Escherichia coli*; OR = odds ratio.

The distribution of the characterized virulence genes varied across the age strata. In 0- to 11-month stratum, prevalence of *pet* (OR: 6.9, 95% CI: 2.06–29.20, *P* < 0.001), *aggA* (OR: 2.2, 95% CI: 1.16–4.29, *P* = 0.008), and *capU* (OR: 1.9, 95% CI: 1.02–3.51, *P* = 0.028) genes were more common in cases compared with controls (Table 3). Similar higher prevalence pattern was observed for *pet* (OR: 15.0, 95% CI: 1.35–750.0, *P* = 0.003) and *capU* (OR: 4.3, 95% CI: 1.27–18.54, *P* = 0.009) when the virulence factors were characterized among the sole EAEC pathogen from MSD children 0- to 11-month age in cases than controls.

**Table 3:** Distribution of EAEC virulence genes in case and control children in three age strata

Virulence genes	0–11 months (*N* = 217)				12–23 months (*N* = 166)				24–59 months (*N* = 45)			
	Case (*N* = 85) no. (%)	Control (*N* = 132) no. (%)	OR (95% CI)	*P* value	Case (*N* = 61) no. (%)	Control (*N* = 105) no. (%)	OR (95% CI)	*P* value	Case (*N* = 11) no. (%)	Control (*N* = 34) no. (%)	OR (95% CI)	*P* value
***aatA***	33 (38.8)	48 (36.4)	1.1 (0.60–2.02)	0.714	16 (26.2)	26 (24.8)	1.1 (0.48–2.34)	0.833	2 (18.2)	8 (23.5)	0.7 (0.06–4.71)	0.710
***aggR***	61 (71.8)	82 (62.1)	1.5 (0.82–2.93)	0.143	33 (54.1)	62 (59.1)	0.8 (0.41–1.62)	0.534	3 (27.3)	17 (50.0)	0.4 (0.05–1.95)	0.187
***aaP***	48 (56.5)	59 (44.7)	1.6 (0.89–2.88)	0.090	28 (45.9)	46 (43.8)	1.1 (0.54–2.15)	0.793	1 (9.1)	16 (47.1)	0.1 (0.00–0.98)	0.024
**ORF3**	64 (75.3)	84 (63.6)	1.7 (0.91–3.37)	0.071	37 (60.7)	63 (60.0)	1.0 (0.51–2.06)	0.933	5 (45.5)	21 (61.8)	0.5 (0.10–2.53)	0.341
***capU***	59 (69.4)	72 (54.6)	1.9 (1.02–3.51)	0.028	42 (68.9)	62 (59.1)	1.5 (0.75–3.18)	0.208	7 (63.6)	24 (70.6)	0.7 (0.14–4.20	0.665
**aar**	62 (72.9)	98 (74.2)	0.9 (0.48–1.82)	0.831	42 (68.9)	70. (66.7)	1.1 (0.53–2.32)	0.772	6 (54.6)	20 (58.8)	0.8 (0.17–4.24)	0.802
***aafC***	3 (3.5)	7 (5.3)	0.7 (0.10–2.96)	0.543	3 (4.9)	6 (5.7)	0.9 (0.13–4.18)	0.827	1 (9.1)	3 (8.8)	1.0 (0.01–14.6)	0.978
***agg3/4C***	34 (40.0)	51 (38.6)	1.1 (0.58–1.91)	0.840	20 (32.8)	33 (31.4)	1.1 (0.50–2.19)	0.856	4 (36.4)	12 (35.3)	1.0 (0.18–5.18)	0.948
***agg3A***	3 (3.5)	18 (13.6)	0.2 (0.04–0.83)	0.014	5 (8.2)	8 (7.6)	1.1 (0.26–3.96)	0.893	2 (18.2)	2 (5.9)	3.6 (0.22–53.6)	0.212
***aafA***	1 (1.2)	3 (2.3)	0.3 (0.01–6.51)	0.557	2 (3.3)	10 (9.5)	0.3 (0.03–1.59)	0.134	0 (0)	2 (5.9)	0.0 (0.00–16.8)	0.410
***aggA***	32 (37.7)	28 (21.2)	2.2 (1.16–4.29)	0.008	13 (21.3)	25 (23.8)	0.9 (0.37–1.95)	0.711	2 (18.2)	9 (26.5)	0.6 (0.05–3.94)	0.578
***agg4A***	10 (11.8)	12 (9.1)	1.3 (0.48–3.55)	0.524	4 (6.6)	2 (1.9)	3.6 (0.49–40.7)	0.121	1 (9.1)	2 (5.9)	1.6 (0.02–33.4)	0.710
***astA***	46 (54.1)	55 (41.7)	1.7 (0.91–2.96)	0.072	41 (67.2)	52 (49.5)	2.1 (1.03–4.27)	0.026	4 (36.4)	22 (64.7)	0.3 (0.05–1.56)	0.098
***sat***	20 (23.5)	24 (18.2)	1.4 (0.66–2.84)	0.338	9 (14.8)	24 (22.9)	0.6 (0.22–1.43)	0.207	0 (0)	8 (23.5)	0.0 (0.00–1.69)	0.076
***sepA***	31 (36.5)	35 (26.5)	1.6 (0.84–2.86)	0.119	16 (26.2)	23 (21.9)	1.3 (0.56–2.79)	0.526	3 (27.3)	4 (11.8)	2.8 (0.33–20.1)	0.217
***pet***	15 (17.7)	4 (3.0)	6.9 (2.06–29.20)	< 0.001	9 (14.8)	16 (15.2)	1.0 (0.34–2.51)	0.933	0 (0)	4 (11.8)	0.0 (0.00–4.78)	0.233
***pic***	24 (28.2)	34 (25.8)	1.1 (0.58–2.18)	0.687	28 (45.9)	41 (39.1)	1.3 (0.66–2.63)	0.387	3 (27.3)	13 (38.2)	0.6 (0.08–3.18)	0.509
***sigA***	4 (4.7)	5 (3.8)	1.3 (0.24–6.01)	0.740	10 (16.4)	20 (19.1)	0.8 (0.32–2.04)	0.668	4 (36.4)	6 (17.7)	2.7 (0.42–15.0)	0.194
***aaiC***	22 (25.9)	34 (25.8)	1.0 (0.51–1.95)	0.983	19 (31.2)	48 (45.7)	0.5 (0.25–1.09)	0.065	3 (27.3)	15 (44.1)	0.5 (0.07–2.47)	0.321
***air***	26 (30.6)	34 (25.8)	1.3 (0.66–2.32)	0.437	13 (21.3)	21 (20.0)	1.1 (0.45–2.50)	0.840	2 (18.2)	2 (5.9)	3.6 (0.22–53.6)	0.212
***eilA***	37 (43.5)	54 (40.9)	1.1 (0.61–2.00)	0.702	35 (57.4)	52 (49.5)	1.4 (0.69–2.72)	0.328	7 (63.6)	22 (64.7)	1.0 (0.19–5.38)	0.948

CI = confidence interval; EAEC = enteroaggregaive *Escherichia coli*; OR = odds ratio.

Prevalence of virulence genes that were proportionately higher in cases compared with controls in children 0–11 months were *sepA* (36.5% versus 26.5%), *astA* (54.1% versus 41.7%), *aggR* (71.8% versus 62.1%), *aap* (56.5% versus 44.7%), and ORF3 (75.3% versus 63.6%). The *astA* gene was found more often in cases (67.2%) than in controls (49.5%) in the age stratum 12–23 months (OR: 2.1, 95% CI1.03–4.27, *P* = 0.026); none of the putative virulence factors were found to be significantly more common in MSD children ≥ 2 years of age (Table 3).

In addition to considering each factor individually, we considered the importance of combinations of potential EAEC virulence factors by employing CART analysis. The CART analysis builds a model in stepwise fashion to yield the combination of factors most strongly associated with the queried outcome, in this case the combination of factors most strongly associated with MSD. Each branch of a CART output tree ends in a terminal “node”; each observation falls into exactly one terminal node; and each terminal node is uniquely defined by a set of rules, such as having or not having a certain factor.

We examined all genotypic assays performed: *aatA*, *aggR*, *aaiC*, *aap*, ORF3, *sat*, *sepA*, *pic*, *sigA*, *pet*, *astA*, *aafC*, *agg3/4C*, *aafA*, *agg3A*, *aggA*, *agg4A*, *air*, *capU*, *eilA*, *aar*, as well as considering the collective number of virulence loci present (generating a VFS) (Supplemental Figures 1 and 2).

As noted, prevalence of the virulence were significantly higher in cases compared with controls in children 0–11 months and applying the CART analysis (Supplemental Figure 2) showed that the presence of *pet* (Node 1), regardless of the presence or absence of any other scored genotype among the *pet*-positive strains, provided a strong association with diarrhea. Among the *pet*-negative strains, CART analysis suggested two additional trait clusters that were associated with MSD: Node 2 includes those strains with a VFS ≤ 8 in combination with *sepA*, whereas Node 3 includes a VFS > 8, suggesting a combination of typical EAEC factors in addition to the toxin EAST-1 toxin.

### Antibiotic susceptibility testing.

The susceptibility patterns of the randomly selected 88 (20%) EAEC strains were similar among cases and controls. The data showed that all the randomly selected 88 (100%) EAEC strains tested were susceptible to ceftriaxone and cefoxitin, over 90% were susceptible to cefotazidime and ciprofloxacin, and more than 80% were susceptible to chloramphenicol and nalidixic acid. However, susceptibility to sulphamethoxazole-trimethoprim and ampicillin was low (15% and 28%, respectively) (Table 4).

**Table 4:** Antimicrobial-resistant pattern of EAEC strains (*N* = 88)

Antibiotics (μg)	Resistant no. (%)	Susceptible no. (%)
Sulphamethoxazole-trimethoprim (25)	75 (85.0)	13 (15.0)
Ampicillin (10)	63 (72.0)	25 (28.0)
Chloramphenicol (30)	16 (18.2)	72 (81.8)
Nalidixic acid (30)	16 (18.2)	72 (81.8)
Ciprofloxacin (5)	8 (9.0)	80 (91.0)
Cefotazidime (30)	2 (2.3)	86 (97.7)
Ceftriaxone (30)	0 (0)	88 (100)
Cefoxitin (30)	0 (0)	88 (100)

EAEC = enteroaggregaive *Escherichia coli*.

## DISCUSSION

EAEC is a common cause of diarrhea worldwide.^32^ The assessment of the 21 genes in the 428 EAEC strains in this study showed that the frequency of most genes correlated well with similar studies, particularly the study from the GEMS neighboring site, in Bamako Mali.^28^ In this study, more than half of participants were younger than 1 year of age, although there were no differences between cases and controls. Particularly, striking was the consistency of the association between SepA and MSD in Mali and this study. SepA is a SPATE protease that was initially found in *Shigella flexneri* strains,^33^ but has subsequently been found commonly among EAEC.^34^ The protease has been implicated in causing increased inflammation in Shigella strains but it may also have enterotoxic activity.

In this study, the virulence genes *aggA* encoding for AAF/1, capU, and pet, encoding a member of Class 1 SPATEs family, were statistically implicated as genes responsible for EAEC diarrhea in younger children < 12 months.

Our study highlights significant heterogeneity in gene profiles among the EAEC isolates. Of the 21 genes targeted, none of the EAEC isolates characterized genetically harbors more than 15 genes. The heterogeneous nature of EAEC enables it to display variation in causing clinical illness,^32^ although factors responsible for its virulence are not well understood.

Several studies have shown possible genes that confer virulence on EAEC.^32,35^ Our data show three virulence genes associated with diarrhea in infants. Interestingly, the three incriminated virulence genes are plasmid genes that include *pet*, AAF/1 fimbrial subunit (*aggA*), and hexosyltransferase homolog (*capU*). The Pet toxin is a 108-kDa protease implicated in cytoskeletal changes and epithelial-cell rounding by cleavage of the cytoskeletal protein spectrin.^36–39^ In Mexico, the Pet gene was initially detected from EAEC strain 049766 implicated in a highly virulent outbreak of diarrhea in which some infants died.^40^ Also, the reported enterotoxic activity of EAEC induced by Pet is consistent with the secretory diarrhea seen in most patients with EAEC enteritis.^41^ A recent report from Iran alluded that pet gene is more prevalent among EAEC strains isolated from adult diarrheal patients.^42^ Therefore, our findings support the role of Pet gene in EAEC causing diarrhea in infants (Supplemental Figure 1; Table 3). However, earlier EAEC virulence factor study conducted in southwest Nigeria over a decade ago showed that the Pet gene was equally distributed among EAEC strains isolated from children < 5 years with or without diarrheal.^43^ Seemingly, our study also showed, no association of Pet with diarrheal disease in the children < 5 years but the effect is only seemed in EAEC strains isolated from children < 1 year and so the differences between our findings could be due to age stratification.

AAF/I was associated with diarrhea in the first year of life. The Shiga toxin producing EAEC strain implicated in the German outbreak expressed AAF/I.^44^

Hexosyltransferase homolog (*capU*), a plasmid-encoded protein was significantly high among the younger children. Its role in EAEC diarrhea is not clearly defined. Notably, the *capU* gene was the third most common gene found (62%) among genes investigated in this study. This probably highlights the importance of genes acting in concert.

*astA* encodes an EAST1 that is related to the heat-labile enterotoxin of enterotoxigenic *E. coli*. The relevance of *astA* gene in EAEC diarrhea has been reported in several studies,^45–48^ and EAST1 was found to be associated with diarrhea in combination with other genes in the Mali study.^28^ *astA* is not restricted to EAEC but is widely distributed among other enteric pathogens^49,50^ as well as commensal *E. coli*.

Globally, EAEC strains have shown a low to high level of resistance to antimicrobial agents.^51^ Our data from the antimicrobial susceptibility investigation highlights resistant pattern of the EAEC strains against cotrimoxazole and ampicillin. The first line of antibiotics prescribed for patient management in our region is cotrimoxazole and ampicillin, which explains the high resistance against these antibiotics. An increase in resistance of EAEC strains to chloramphenicol, nalidixic acid, and quinolones was observed in this study compared with a similar study on a member of enterobacteriaceae family from the same region^52^ and in eastern Asia.^53^ Twenty percent of the EAEC strains tested showed multidrug resistance to three antimicrobial agents, whereas 6% showed resistance to more than three antimicrobial agents. This finding is in contrast to a similar study conducted in India, showing 75% of strains with multidrug resistance, that is, > 3 antimicrobial agents.^54^

The limitations of this study included exclusion of multiple comparisons such as malnutrition and other enteric coinfections. Hence, future studies can consider these essential confounders.

Our study has strengthened the role of *pet* and *aggA* genes of EAEC in the cause of MSD in African infants. The EAEC virulence gene profiles found in this study have also proven the heterogeneity of the genetic component of the EAEC isolates studied. However, further investigations are needed to establish the specific or combination of gene(s) that are associated with EAEC diarrheal in different age strata, particularly children from developing countries. In addition, the pattern of antimicrobial resistance against EAEC is worrisome and needs to be addressed.

## Supplementary Material

Supplemental Figure.
