# Supplementary material for: Identification of Subsets of Enteroaggregative Escherichia coli Associated with Diarrheal Disease among Under 5 Years of Age Children from Rural Gambia
Source: Am J Trop Med Hyg. 2017 Aug 7;97(4):997–1004. doi: 10.4269/ajtmh.16-0705 (PMC5637583; doi:10.4269/ajtmh.16-0705)
Supplement: Supplementary file 1 [file tpmd160705.SD1.pdf]

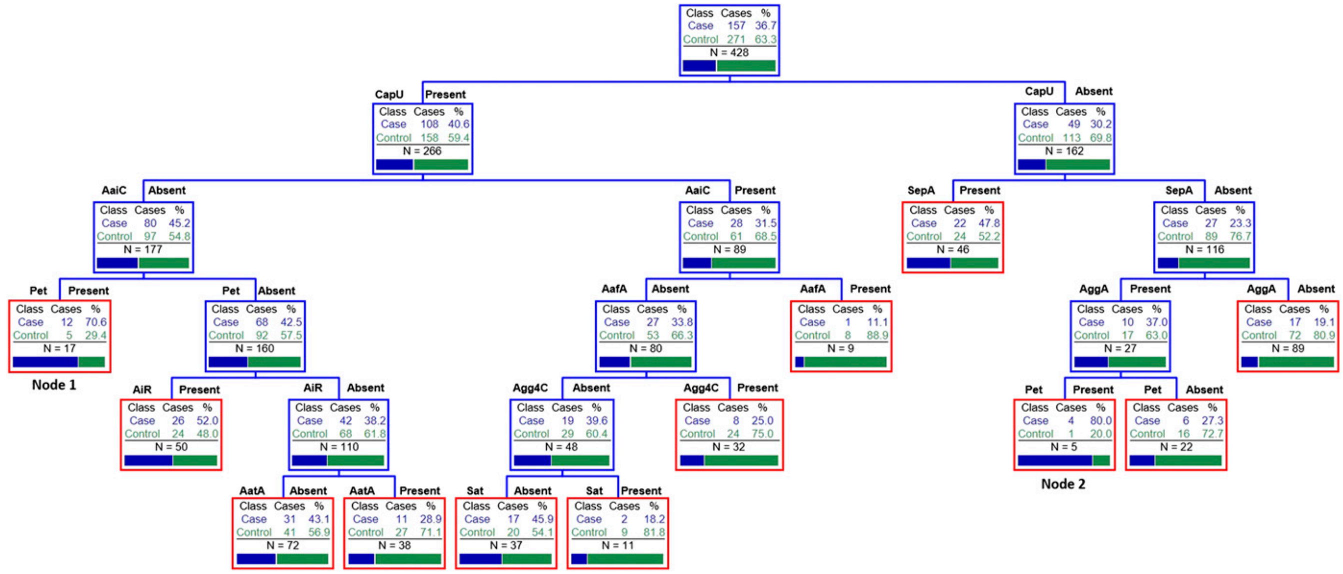

Supplemental Figure 1. Association of virulence factors with diarrhea in children aged 0–59 months. Each branch of the Classification and Regression Tree ends in a terminal “node” (red boxes), and each terminal node is uniquely defined by the presence or absence of a predictive factor such as a gene or virulence factor score.

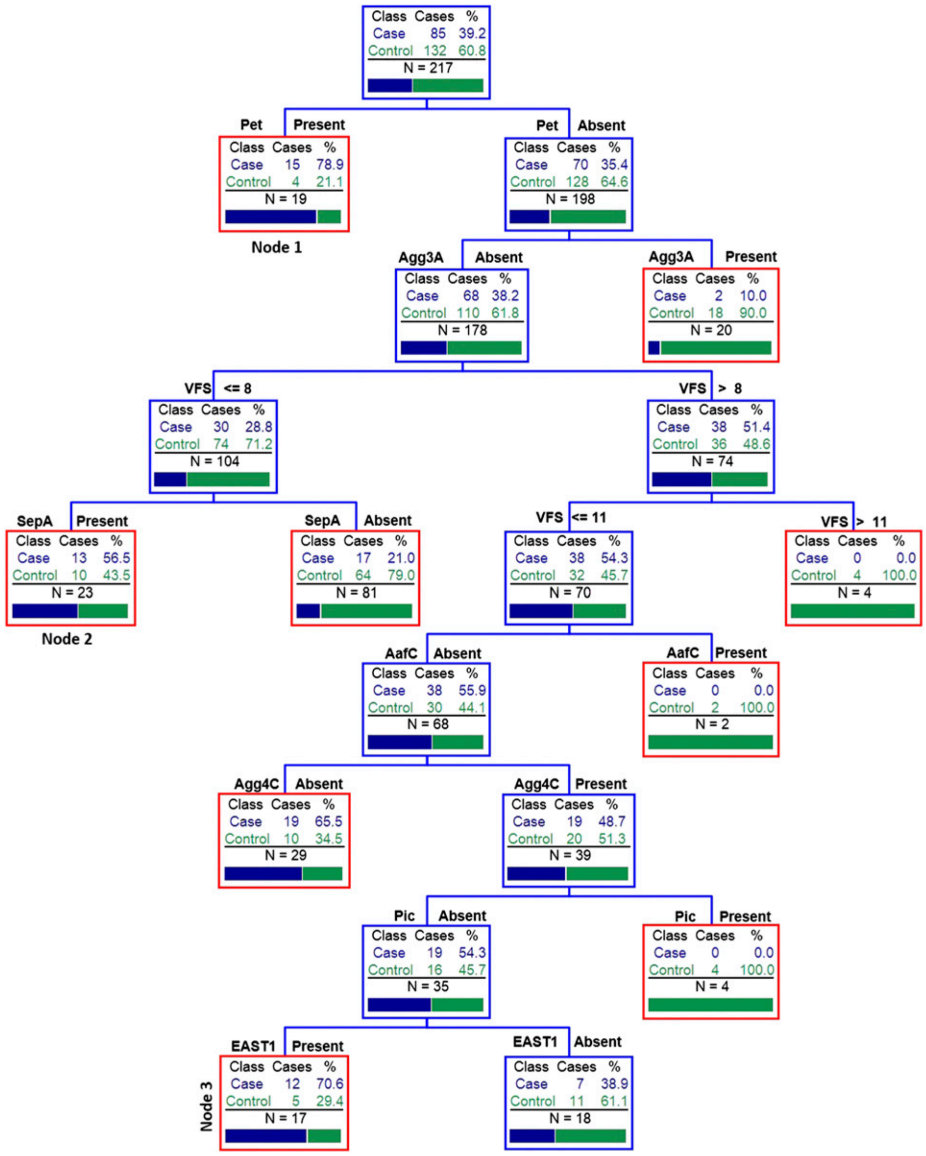

Supplemental Figure 2. Association of virulence factors with diarrhea in children aged 0–11 months. Each branch of the Classification and Regression Tree ends in a terminal “node” (red boxes), and each terminal node is uniquely defined by the presence or absence of a predictive factor such as a gene or virulence factor score (VFS).
